# Bridging Gaps in Occupational Respiratory Disease Management: A Comparative Survey of Pulmonologists and Occupational Physicians in Italy

**DOI:** 10.3390/clinpract15100174

**Published:** 2025-09-24

**Authors:** Alessandra Tortorella, Alessio Marinelli, Luigi De Maria, Silvano Dragonieri, Giuseppe Del Vecchio, Vitaliano Nicola Quaranta, Andrea Portacci, Giovanna Elisiana Carpagnano, Luigi Vimercati

**Affiliations:** 1 Department of Respiratory Diseases, University of Bari, 70121 Bari, Italy; a.tortorella7@studenti.uniba.it (A.T.); alessio.marinelli@uniba.it (A.M.); vitalianonicola.40@gmail.com (V.N.Q.); a.portacci01@gmail.com (A.P.); elisiana.carpagnano@uniba.it (G.E.C.); 2 Department of Occupational Medicine, University of Bari, 70121 Bari, Italy; luigi.demaria@uniba.it (L.D.M.); giuseppe.delvecchio1@uniba.it (G.D.V.); luigi.vimercati@uniba.it (L.V.)

**Keywords:** occupational respiratory diseases, interdisciplinary collaboration, pulmonologists, occupational physicians

## Abstract

**Background**: Themanagement of occupational respiratory diseases (ORDs) requires a multidisciplinary approach, yet collaboration between pulmonologists and occupational physicians is often fragmented, potentially compromising patient outcomes. This study aimed to systematically compare the management strategies for ORDs between these two specialties in Italy to identify gaps and opportunities for integration. **Methods**: A cross-sectional survey was conducted using a structured 12-item questionnaire distributed to board-certified pulmonologists and occupational physicians across Italy. The questionnaire assessed diagnostic pathways, therapeutic strategies, preventive measures, and patterns of interdisciplinary collaboration. A total of 102 specialists (51 pulmonologists and 51 occupational physicians) completed the survey. Comparative analyses were performed using Pearson’s $\chi^{2}$ tests. **Results**: Significant divergences in practice were identified. Pulmonologists primarily focused on clinical diagnosis, utilizing pulmonary function tests (34.3%) and imaging (11.8%), and favored pharmacotherapy (27.5%) as the first-line treatment, in alignment with clinical guidelines. Conversely, occupational physicians prioritized detailed occupational and exposure histories (15.7%) and preventive interventions aimed at exposure reduction (15.7%). While both groups acknowledged the importance of collaboration, a substantial number reported that it occurred only occasionally (17.6% of pulmonologists and 12.7% of occupational physicians), indicating a significant gap in integrated care. Shared barriers included poor patient adherence and limited access to advanced diagnostic tools. **Conclusions**: While sharing a common foundation in diagnostic and preventive principles, pulmonologists and occupational physicians in Italy operate with distinct, complementary approaches that remain insufficiently integrated. The observed fragmentation in diagnostic and therapeutic pathways underscores an urgent need for shared national guidelines, structured interdisciplinary training, and formalized communication protocols. Bridging this disciplinary divide is essential to delivering holistic care, optimizing worker health, and preserving work ability.

## 1. Introduction

Occupational respiratory diseases (ORDs) represent a diverse group of acute and chronic conditions, such as occupational asthma and pneumoconioses, caused or exacerbated by workplace exposures. These include occupational asthma, chronic obstructive pulmonary disease (COPD) linked to dust, fume, or gas exposure, hypersensitivity pneumonitis due to organic dusts, and pneumoconioses such as asbestosis, silicosis, and coal workers’ pneumoconiosis [1,2]. This is not a localized issue but a significant global health problem, accounting for substantial morbidity, disability-adjusted life years lost, and economic burden due to absenteeism, presenteeism, and compensation costs [3].

ORDs are caused by inhalation of noxious agents leading to inflammatory, allergic, or fibrotic reactions within the respiratory tract [4]. The pathophysiology varies by disease: Asthma involves eosinophilic or neutrophilic airway inflammation and hyperresponsiveness; COPD results from chronic exposure to irritants causing airway remodeling and parenchymal destruction; and pneumoconioses feature deposition of inorganic dusts triggering macrophage activation and fibrosis [5]. Early identification and intervention are critical to prevent irreversible lung damage, progressive functional decline, and socioeconomic consequences [6].

The recognition of ORDs dates to antiquity, culminating in Bernardino Ramazzini’s systematic work in 1700 [7]. Nevertheless, modern management still fundamentally requires timely exposure recognition, accurate diagnosis, appropriate treatment, and preventive interventions [8].

The management of ORDs is inherently multidisciplinary. Pulmonologists focus on clinical diagnosis, functional assessment, and pharmacotherapy according to guidelines such as Global Initiative for Asthma (GINA) and Global Initiative for Chronic Obstructive Lung Disease (GOLD) [9,10]. Occupational physicians assess exposure histories, perform workplace risk evaluations, recommend preventive measures, and manage medico-legal aspects such as disease certification and work ability assessment [11]. However, a lack of structured collaboration between these professionals may result in delayed diagnosis, incomplete exposure mitigation, fragmented care, and suboptimal health outcomes [12].

Existing literature suggests that integrated care models, combining clinical management with workplace interventions, lead to improved disease control, reduced exacerbations, and preserved work capacity [13].

In Italy, despite advances in occupational safety, ORDs remain a public health priority. The urgency is underscored by several converging factors. First, according to Istituto Nazionale Assicurazione contro gli Infortuni sul Lavoro (INAIL) data, there was a 21.6% increase in all occupational disease claims in 2024 compared to 2023, with respiratory diseases accounting for 2.5% of all claims [14]. Second, this official figure likely underestimates the true burden due to significant gaps in surveillance, including underreporting, misclassification, and a lack of standardized systems [15]. This data vacuum directly contributes to the fragmented clinical approaches this study investigates, creating an immediate public health imperative to establish integrated care pathways and robust, evidence-based national guidelines. This gap creates a significant void in evidence, directly impacting clinical practice by fostering the regional and disciplinary variations in management that this study investigates. Without a clear epidemiological picture, practitioners are left to navigate diagnosis and prevention without a unified, data-driven national strategy. The challenges identified within the Italian system, such as fragmented care and the need for better collaboration between clinical and occupational specialists, are not unique. These issues mirror findings from broader European studies, which also highlight a systemic gap between clinical diagnosis and occupational exposure assessment.

This gap in knowledge gives rise to this study’s primary research question:

What are the key differences and similarities in the diagnostic pathways, therapeutic strategies, and preventive approaches for ORDs between pulmonologists and occupational physicians in Italy? By answering this question, we aim to identify crucial areas for integration that can inform the development of shared guidelines, training curricula, and policy interventions to optimize the health and work ability of exposed workers.

## 2. Materials and Methods

### 2.1. Study Design and Objectives

This study employed a cross-sectional observational design to systematically assess and compare the diagnostic, therapeutic, and preventive practices of pulmonologists and occupational physicians in Italy in managing occupational respiratory diseases (ORDs). The primary objective was to identify differences, similarities, and potential areas for integrated management to inform future shared guidelines and training curricula.

### 2.2. Study Population and Recruitment

The study population consisted of board-certified pulmonologists and occupational physicians practicing in Italy. Eligible participants were identified through professional societies, including the Italian Respiratory Society (SIP/IRS) and the Italian Society of Occupational Medicine (SIML), as well as institutional and academic networks.

Between January and March 2025, email invitations containing study information and a secure electronic survey link were distributed.

Inclusion criteria were as follows:

1. Current active clinical practice as a pulmonologist or occupational physician in Italy;
2. Willingness to participate voluntarily;
3. Completion of the entire questionnaire.

Exclusion criteria included retired physicians and incomplete survey responses.

A total of 102 physicians participated, comprising 51 pulmonologists and 51 occupational physicians, achieving a balanced sample representative of northern, central, and southern Italian regions.

### 2.3. Questionnaire Development

A structured 12-item questionnaire was developed based on an extensive literature review and adapted to reflect the core diagnostic, therapeutic, and preventive principles outlined in major evidence-based guidelines on occupational asthma and ORD management [1,2]. The questionnaire was designed to capture real-world diagnostic approaches, therapeutic strategies, preventive measures, interdisciplinary collaboration patterns, and perceived systemic barriers.

Content validity and clarity were ensured through expert panel review involving three pulmonologists, three occupational physicians, and two public health researchers. Minor modifications were made based on feedback to enhance question clarity and relevance.

### 2.4. Survey Content

The final questionnaire covered the following domains:

1. **Initial evaluation methods** for suspected ORDs (e.g., medical history, occupational exposure history, spirometry, imaging);
2. **Diagnostic tools** routinely used;
3. **Diagnostic criteria** applied for ORD confirmation;
4. **First-line therapeutic strategies**, including pharmacological and non-pharmacological interventions;
5. **Frequency and structure of follow-up visits;**
6. **Preventive interventions** recommended to patients and employers;
7. **Frequency and modality of interdisciplinary collaboration;**
8. **Patient education topics** addressed during consultations;
9. **Tools and methods for monitoring disease progression;**
10. **Perceived systemic and operational barriers** to optimal ORD management;
11. **Use of national or international guidelines** in clinical practice;
12. **Perceived priorities** for improving ORD management in Italy.

Most items were multiple-choice with optional free-text fields for elaboration. To better elucidate the distinct priorities of each specialty, several multiple-choice questions were designed to be mutually exclusive, requiring respondents to select the single best option that represented their primary or first-line approach. Therefore, the resulting percentages reflect the distribution of these prioritized choices rather than the complete range of all actions a physician might take.

### 2.5. Data Collection Procedures

Surveys were administered electronically using the secure University of Bari REDCap platform to ensure data confidentiality and ease of completion. Two follow-up reminder emails were sent at two-week intervals to maximize response rates. Responses were anonymized upon submission and stored in an encrypted institutional database accessible only to the principal investigator and study statistician.

### 2.6. Statistical Analysis

All data were analyzed using R Core Team (2025) [16] and RStudio (v. 2025.5.1.513) [17]. Descriptive statistics, including frequencies, percentages, means, and standard deviations, summarized participant characteristics and response distributions. The hypotheses for the test are as follows:

- H0 (null hypothesis): Variable 1 is independent of Variable 2.
- H1 (alternative hypothesis): Variable 1 is not independent of Variable 2.

To compare the responses to categorical variables between the two groups of specialists (pulmonologists and occupational physicians), Pearson’s $\chi^{2}$ test was employed. The validity of this test is contingent upon adhering to Cochran’s rule, which states that no more than 20% of the cells in a contingency table should have an expected frequency of less than 5. Therefore, a preliminary check of this assumption was performed for each analysis.

In cases where this condition was not met, indicating that the $\chi^{2}$ approximation could be inaccurate, Fisher’s exact test was used instead. This alternative approach calculates the exact probability and does not depend on minimum frequency assumptions, thus ensuring more robust and reliable results, especially in the presence of sparse data or small sample sizes. For all analyses, a *p*-value of <0.05 was considered statistically significant (Table 1). For all statistically significant associations, Cramér’s V was calculated as a measure of effect size to evaluate the strength and clinical relevance of the relationship. Free-text responses were analyzed qualitatively to identify recurring themes related to perceived barriers and improvement priorities.

A post hoc power analysis was conducted to determine the statistical power of our final sample size. The analysis was based on the $\chi^{2}$ test, which also served as a robust approximation for Fisher’s exact test. It showed that a sample size of approximately 132 participants would be required to reliably detect a medium effect size with 80% power to detect a medium effect size (Cramér’s V > 0.30) at a significance level of 0.05.

## 3. Results

### 3.1. Participant Characteristics

A total of 102 physicians completed the survey, comprising 51 pulmonologists and 51 occupational physicians, achieving a balanced sample (Table 2). Pulmonologists were predominantly hospital-based clinicians (62% in tertiary care hospitals, 28% in secondary-level public hospitals, and 10% in private practice) with a mean age of 47.5 ± 9.2 years and average clinical experience of 18 years. Occupational physicians mainly worked within territorial occupational health units (72%), with 18% employed directly by large industries and 10% in private consultancy, and had a mean age of 50.1 ± 8.4 years with average professional experience of 20 years. Regional representation was evenly distributed across northern, central, and southern Italy.

### 3.2. Initial Evaluation Methods

**Figure 1:**
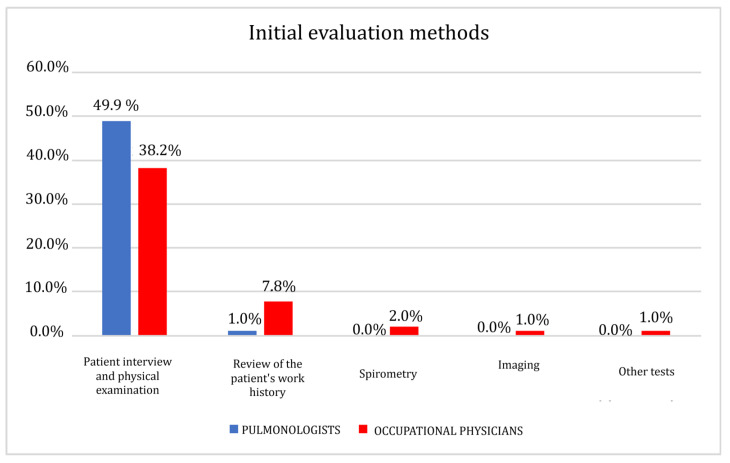
Initial evaluation methods.

Data were analyzed using Fisher’s exact test due to the presence of low expected frequencies. A statistically significant association was found (*p* = 0.004). The strength of this association, as measured by Cramér’s V, indicated a medium effect size (V = 0.325), suggesting a clinically relevant difference between the two specialist groups. The results show a substantial convergence of occupational physicians (38.2%) and pulmonologists (49%) on the patient interview and physical examination as the initial assessment method (Figure 1 and Table 3). Only for a percentage of 7.8% of occupational physicians, the review of the patient’s work history is also significant; on the contrary, a lower percentage of pulmonologists, equal to 1%, considered this method significant in the initial assessment phase.

### 3.3. Diagnostic Tools

**Figure 2:**
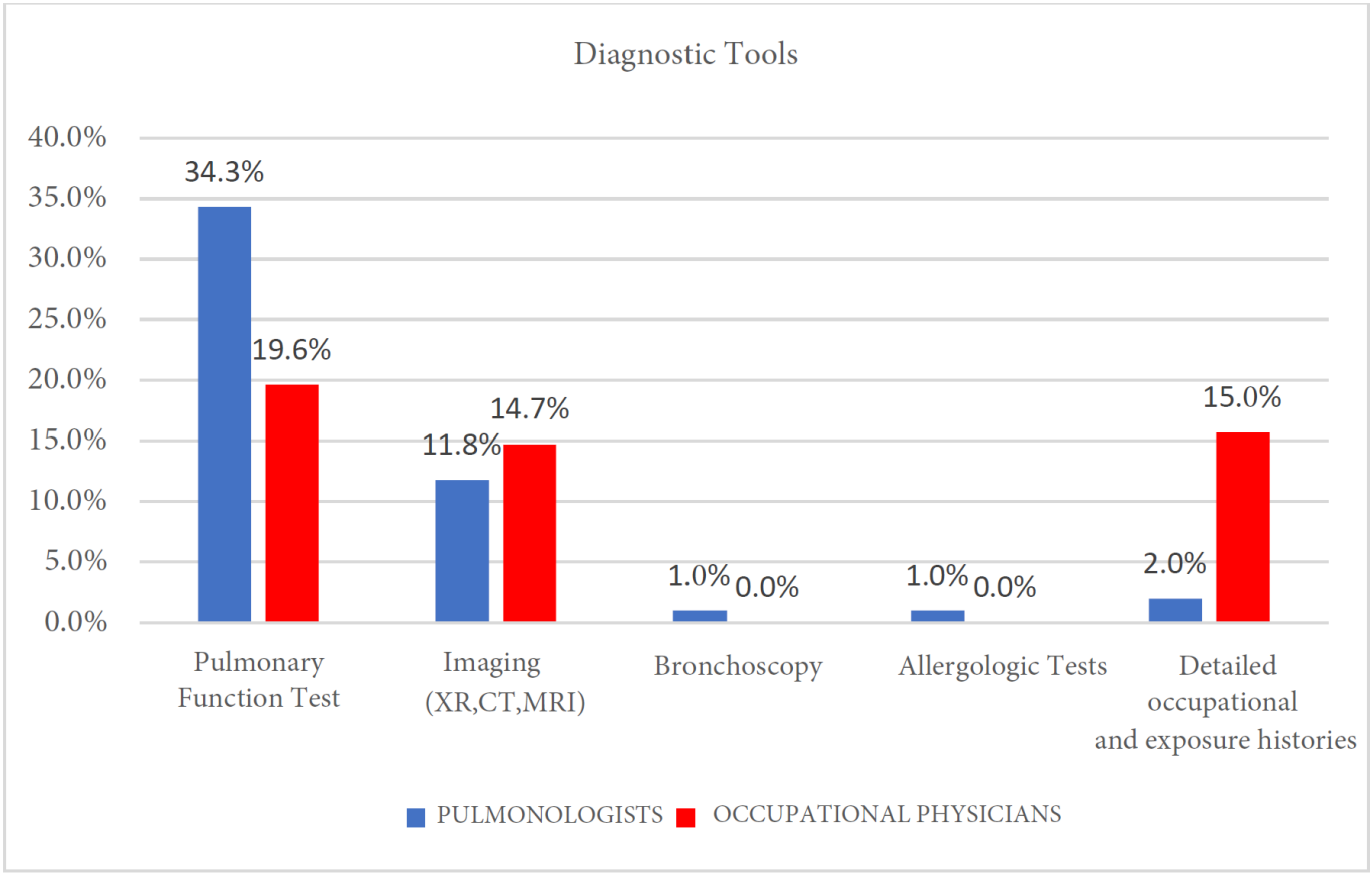
Diagnostic Tools. XR, X-Rays; CT, Computed Tomography; MRI, Magnetic Resonance Imaging.

Data were analyzed using Fisher’s exact test due to the presence of low expected frequencies. A statistically significant association was found (*p* = 0.0003). The strength of this association, as measured by Cramér’s V, indicated a medium effect size (V = 0.412), suggesting a clinically relevant difference between the two specialist groups. While both specialties reported using common diagnostic tools, our analysis revealed significant differences in their primary approaches and frequency of use. A key area of divergence was in the prioritization of functional versus exposure assessment. Pulmonologists reported widespread use of spirometry (34.3%) as the first-line diagnostic tool, mainly for confirming obstructive patterns in suspected occupational asthma or COPD (Figure 2 and Table 4). Imaging techniques, such as chest X-rays and HRCT, were also routinely used by 11.8% of pulmonologists to assess for interstitial changes. Their diagnostic toolkit was further complemented by allergologic tests (1%) and bronchoscopy (1%) in specific clinical scenarios.

In contrast, occupational physicians placed the greatest emphasis on detailed occupational and exposure histories (15.7%), integrating standardized questionnaires with workplace data. Although they also utilize spirometry (19.6%), it is often within the context of periodic health surveillance rather than for primary diagnosis. The use of imaging was comparable between groups, with 14.7% of occupational physicians ordering these studies directly, though many reported referring patients to pulmonologists for radiological evaluation. They made limited use (<1%) of allergologic testing or bronchoscopy, typically deferring these specialized procedures.

### 3.4. Diagnostic Criteria

**Figure 3:**
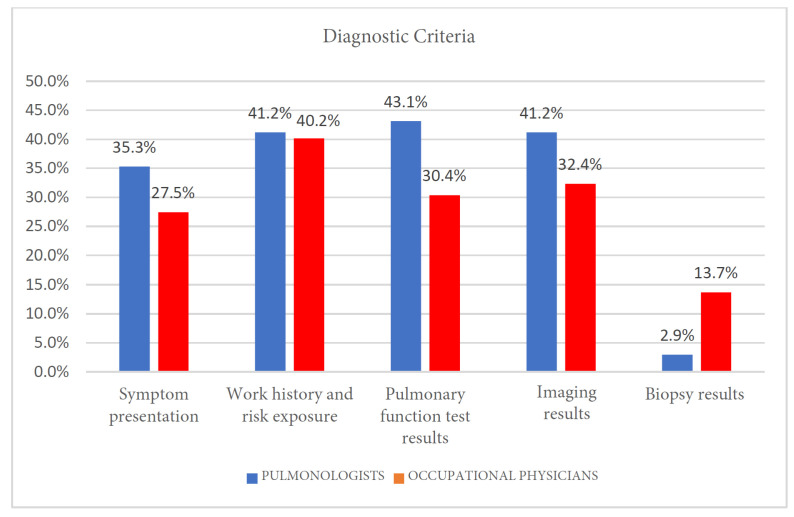
Diagnostic criteria.

Data were analyzed using Pearson’s $\chi^{2}$ test. A statistically significant association was found (*p* = 0.01). The strength of this association, as measured by Cramér’s V, indicated a small to medium effect size (V = 0.201), suggesting that its clinical relevance may be limited. The results show a substantial convergence on work history and risk exposure with 41.2% of pulmonologists and 40.2% of occupational physicians (Figure 3 and Table 5). Pulmonologists place greater emphasis than occupational physicians on symptom presentation (35.3% versus 27.5%), pulmonary function test results (43.1% versus 30.4%), and imaging results (41.2% versus 32.4%). Additionally, biopsy results were considered a relevant diagnostic criterion by a minority of respondents, noted by 13.7% of occupational physicians and 2.9% of pulmonologists.

A notable divergence in diagnostic criteria was the greater reliance on imaging results by pulmonologists. This difference reflects their distinct professional mandates. For a pulmonologist, imaging is paramount for the clinical characterization of lung pathology—identifying patterns of fibrosis or airway disease that are essential for diagnosis and staging according to clinical guidelines. In contrast, the primary role of an occupational physician is to establish a causal link between the disease and workplace exposure. While imaging confirms the presence of a disease, the occupational and exposure history serves as the crucial evidence for determining work-relatedness, which is fundamental for medico-legal assessments and preventive interventions in the workplace.

### 3.5. First-Line Therapeutic Strategies

**Figure 4:**
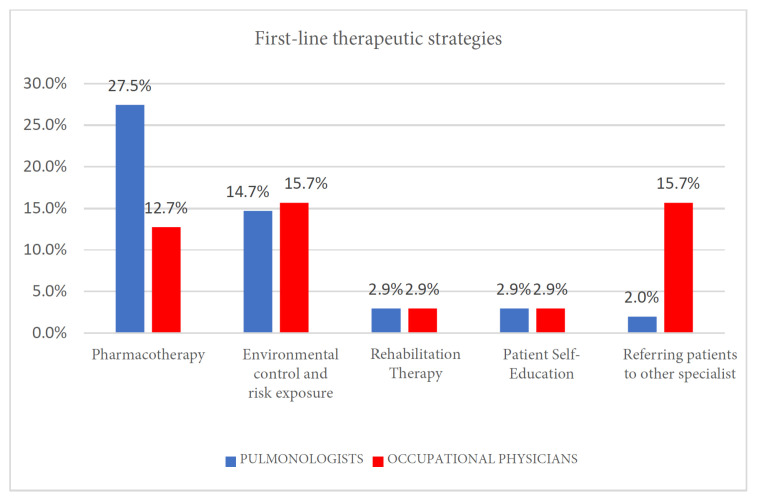
First-line therapeutic strategies.

Data were analyzed using Fisher’s exact test due to the presence of low expected frequencies. A statistically significant association was found (*p* = 0.0001). The strength of this association, as measured by Cramér’s V, indicated a medium effect size (V = 0.401), suggesting a clinically relevant difference between the two specialist groups. Marked differences emerged in therapeutic approaches (Figure 4 and Table 6):

- Pulmonologists:
  - A total of 27.5% initiated pharmacotherapy as first-line management. Treatment regimens included inhaled corticosteroids (ICS) and long-acting beta-agonists (LABA) for asthma, ICS/LABA or triple therapy for COPD, and leukotriene receptor antagonists as add-on therapy for asthma when indicated.
- Occupational physicians:
  - A total of 15.71% prioritized exposure reduction interventions as their primary strategy. Recommendations included workplace relocation, engineering controls, and enhanced PPE use.
  - Only 12.7% directly initiated pharmacotherapy, typically referring patients to pulmonologists for medical management.

### 3.6. Frequency and Structure of Follow-Up Visits

**Figure 5:**
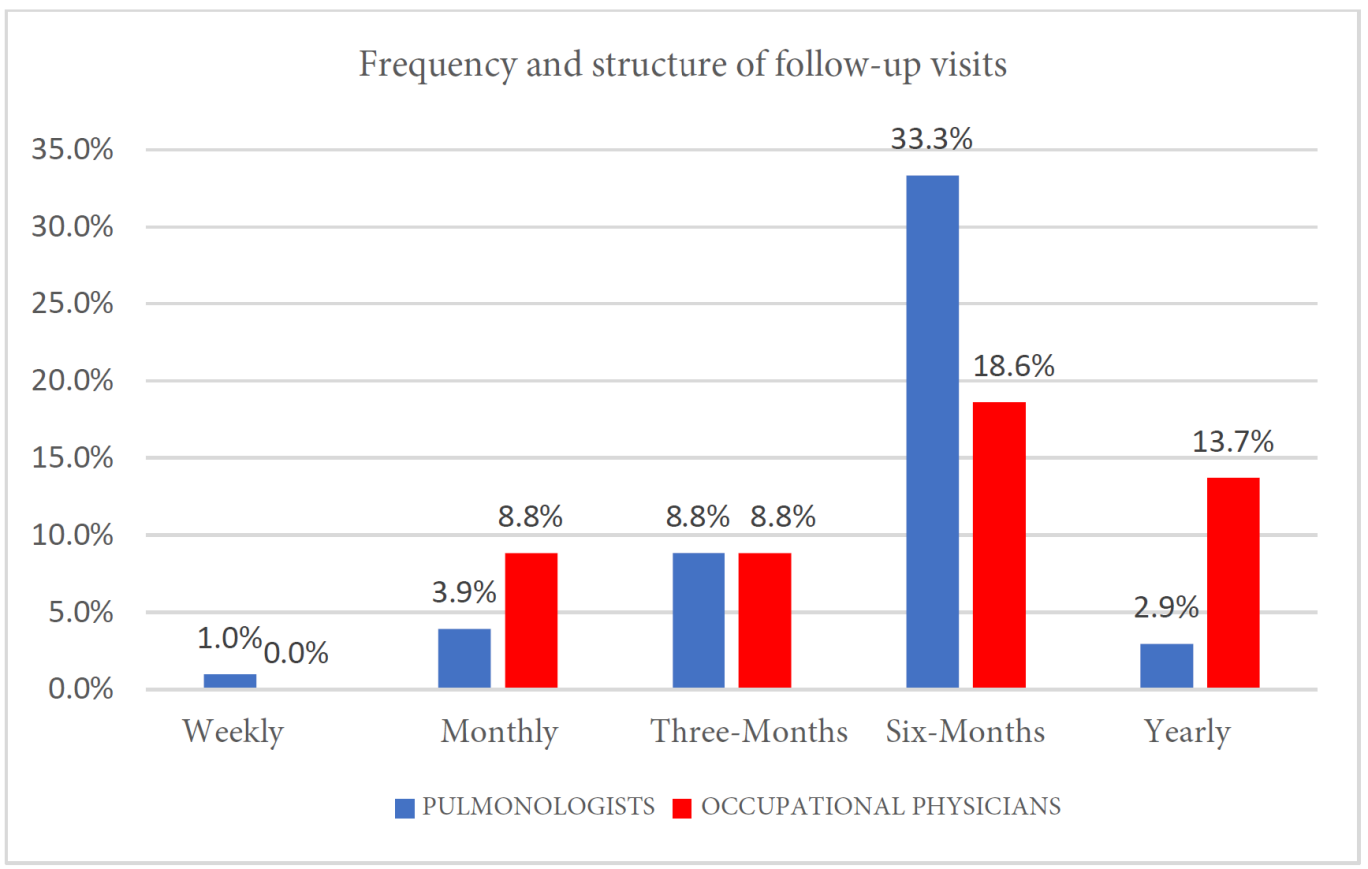
Frequency and structure of follow-up visits.

Data were analyzed using Pearson’s $\chi^{2}$ test. A statistically significant association was found (*p* = 0.0001). The strength of this association, as measured by Cramér’s V, indicated a large effect size (V = 0.841), suggesting a clinically relevant difference between the two specialist groups. Follow-up patterns reflected each specialty’s focus (Figure 5 and Table 7):

- Pulmonologists scheduled visits based on disease severity, with 33.3% reviewing patients every 6 months and 8.8% every 3 months in severe or unstable cases.
- Occupational physicians conducted semestral surveillance visits for most workers (18.6%), increasing frequency for symptomatic workers under evaluation for occupational disease certification or with known ORD diagnoses.

### 3.7. Preventive Interventions Recommended to Patients and Employers

**Figure 6:**
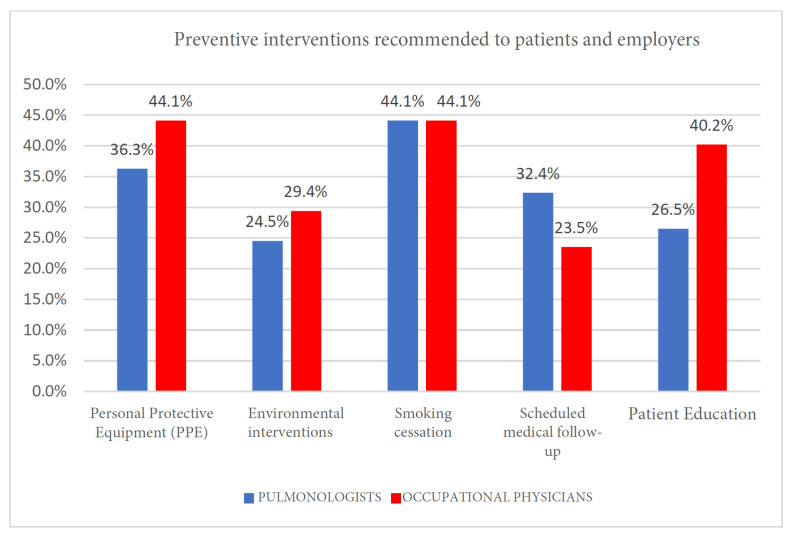
Preventive interventions recommended to patients and employers.

While no statistical significance was reached (Pearson’s $\chi^{2}$ test, *p* = 0.327), we observed both groups endorsing preventive strategies, albeit with slighty differing emphases (Figure 6 and Table 8):

- Smoking cessation programs were equally recommended by both groups (44.1% of pulmonologists and 44.1% of occupational physicians).
- Environmental interventions, such as improved workplace ventilation, substituting hazardous substances, and modifying work processes, were recommended by 29.4% of occupational physicians compared to 24.5% of pulmonologists.

### 3.8. Frequency and Modality of Interdisciplinary Collaboration

**Figure 7:**
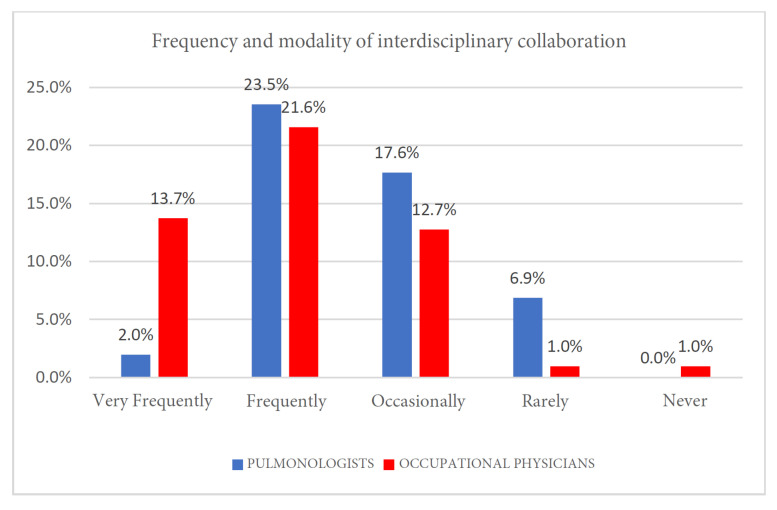
Frequency and modality of interdisciplinary collaboration.

Data were analyzed using Fisher’s exact test due to the presence of low expected frequencies. A statistically significant association was found (*p* = 0.002). The strength of this association, as measured by Cramér’s V, indicated a medium effect size (V = 0.388), suggesting a clinically relevant difference between the two specialist groups. Both specialties indicated that interdisciplinary collaboration occurs with some regularity (Figure 7 and Table 9). The most common response for both pulmonologists and occupational physicians was “Frequently” (23.5% and 21.6%, respectively). However, notable differences emerged across the full spectrum of responses. Occupational physicians were significantly more likely to report collaboration “Very frequently” (13.7%) compared to pulmonologists (2.0%). Conversely, a larger portion of pulmonologists reported that collaboration happens only “Occasionally” (17.6%) or “Rarely” (6.9%).

Collaboration typically occurred in three situations:

- During medico-legal evaluations for disease certification;
- For complex diagnostic cases requiring combined clinical and occupational assessments;
- Through occasional multidisciplinary meetings within hospital or territorial health settings.

Barriers to collaboration included lack of institutional protocols, unclear referral pathways, and limited time.

### 3.9. Patient Education Topics Addressed During Consultations

**Figure 8:**
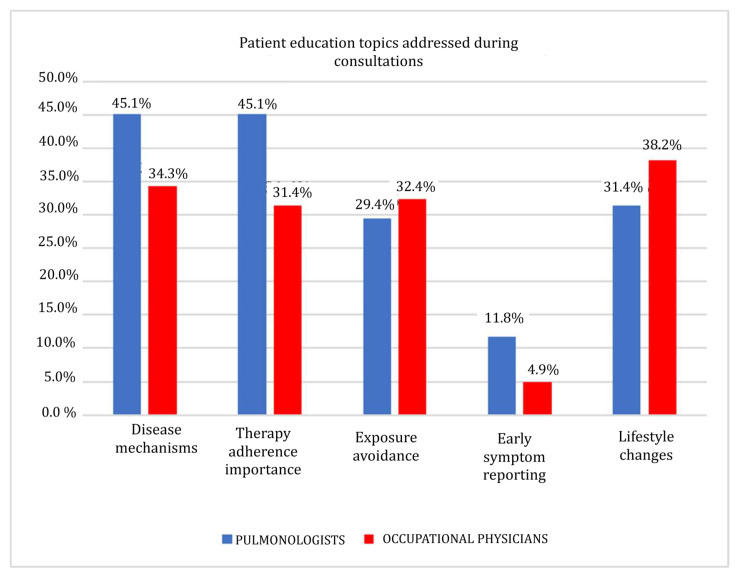
Patient education topics addressed during consultations.

While no statistical significance was reached (Pearson’s $\chi^{2}$ test, *p* = 0.185), both groups were observed to be emphasizing several key educational topics, with some variations in the frequency of their responses (Figure 8 and Table 10):

- Pulmonologists focused on explaining disease mechanisms, pharmacological therapy rationale, inhaler technique, and adherence importance.
- Occupational physicians emphasized recognizing workplace hazards, proper PPE usage, exposure avoidance, and early symptom reporting to facilitate preventive interventions.

### 3.10. Tools and Methods for Monitoring Disease Progression

**Figure 9:**
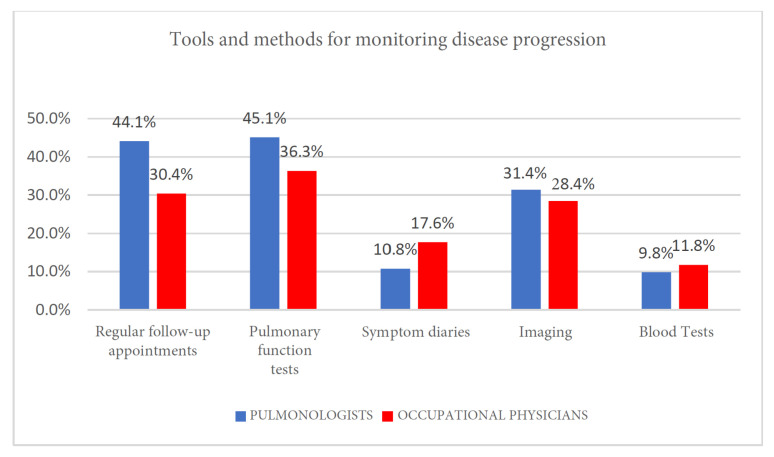
Tools and methods for monitoring disease progression.

No statistical significance was reached (Pearson’s $\chi^{2}$ test, *p* = 0.340). The most selected answers, albeit with different percentages, were pulmonary function tests, as the most utilized tools by both groups (pulmonologists, 45.1%; occupational physicians, 36.3%); regular follow-up appointments (pulmonologists, 44.1%; occupational physicians, 30.4%) and imaging studies (pulmonologists, 31.4%; occupational physicians, 28.4%) (Figure 9 and Table 11).

Since this question in the survey allowed for multiple answers, the examination of the graph shows, beyond the differences highlighted above, a tendency by both categories of specialists to identify methods for monitoring disease progression that are essentially focused on pulmonary function tests, follow-up appointments, and imaging studies.

Symptom diaries and blood tests are considered less adequate methods for monitoring disease progression, although with higher percentage values for occupational physicians compared to pulmonologists.

### 3.11. Perceived Systemic and Operational Barriers to Optimal ORD Management

**Figure 10:**
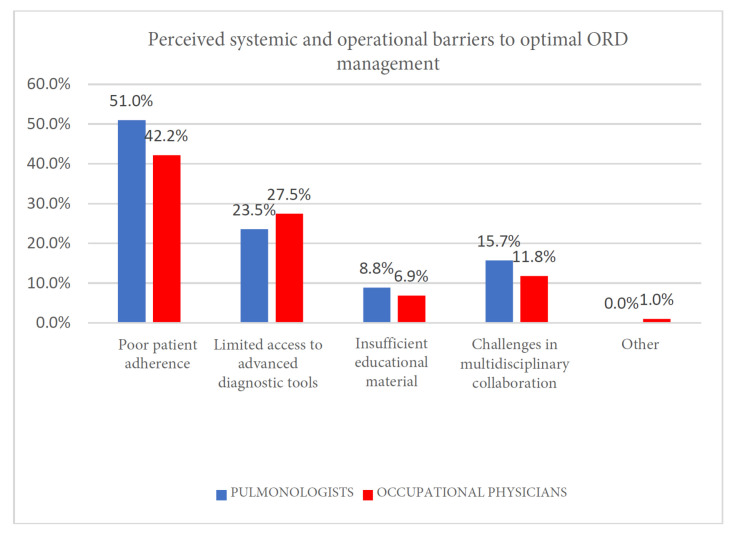
Perceived systemic and operational barriers to optimal ORD management.

While no statistical significal was reached (Pearson’s $\chi^{2}$ test, *p* = 0.650), both specialties were observed to share common barriers (Figure 10 and Table 12):

- Limited access to advanced diagnostic tools (pulmonologists, 23.5%; occupational physicians, 27.5%), particularly in peripheral settings;
- Poor patient adherence to pharmacotherapy or exposure recommendations (pulmonologists, 51%; occupational physicians, 42.2%);
- Challenges in multidisciplinary collaboration integrating clinical and occupational management pathways (pulmonologists, 15.7%; occupational physicians, 11.8%).

### 3.12. Free-Text Responses

Qualitative analysis of free-text responses highlighted recurring themes:

- The need for integrated clinical-occupational guidelines;
- Calls for structured interdisciplinary training and joint workshops to improve mutual understanding of roles and referral pathways.

## 4. Discussion

This nationwide survey reveals that while pulmonologists and occupational physicians in Italy approach ORDs from different perspectives, they operate from a significant shared foundation. This common ground is evident in their mutual endorsement of key preventive measures, such as smoking cessation, and their reliance on core monitoring tools like pulmonary function tests. However, despite this overlap, our findings also highlight that the two specialties apply these tools with statistically significant differences in focus and priority, reflecting their distinct and complementary professional mandates in managing occupational respiratory health.

### 4.1. Diagnostic Approaches

A key finding of this survey is the seemingly low percentage of specialists endorsing fundamental practices like the clinical interview or pharmacotherapy as their primary approach. This should not be interpreted as a deviation from standard medical practice. Rather, it is a direct consequence of the study’s forced-choice methodology, which was designed to reveal the core priorities that differentiate the two specialties. The results powerfully illustrate that while both groups share a common set of tools, their professional mandates—clinical diagnosis for pulmonologists versus causal attribution for occupational physicians—lead them to prioritize different aspects of the clinical encounter. Pulmonologists emphasized clinical diagnostics, using spirometry, imaging, and allergologic testing in line with guideline recommendations for asthma and COPD [9,10]. The high reported use of spirometry underscores their centrality in confirming obstructive or interstitial patterns and guiding treatment decisions. The shared reliance on core methods like spirometry and imaging represents a crucial point of convergence, yet the statistically significant difference in their application highlights the distinct focus of each specialty. Similarly, the reliance of pulmonologists on spirometry aligns with Nicholson et al.’s [15] call for objective functional testing to confirm a diagnosis. Routine use of methacholine challenge tests by pulmonologists for suspected occupational asthma is consistent with the need for objective demonstration of airway hyperresponsiveness in diagnostic algorithms [3] Conversely, occupational physicians prioritized detailed occupational histories and exposure assessments, reflecting their preventive health focus and medico-legal responsibilities in establishing causation. Their relatively lower direct use of imaging and allergologic testing highlights the division of clinical and occupational roles within the Italian health system. Similar diagnostic practice patterns have been reported in European studies, indicating a systemic delineation between exposure assessment and clinical diagnosis [4].

### 4.2. Treatment Strategies

Therapeutic priorities diverged substantially. Pulmonologists focused on pharmacological management, prescribing inhaled corticosteroids and bronchodilators per GINA and GOLD guidelines [9,10], with 25.7% initiating pharmacotherapy as first-line treatment. Occupational physicians, in contrast, emphasized exposure reduction interventions (15.7%) including workplace modifications, engineering controls, and PPE training. This reflects their preventive mandate and the fundamental occupational health principle of removing or reducing exposure as the primary intervention [5]. The limited direct initiation of pharmacotherapy by occupational physicians underscores their reliance on pulmonologists for medical management, while they focus on workplace risk mitigation and surveillance. Furthermore, the approach of occupational physicians directly reflects the primary recommendation from Nicholson et al. [15], which states that for a worker with confirmed occupational asthma, the best chance of recovery is offered by advising them to “avoid further exposure completely and early in the course of their disease”.

### 4.3. Preventive Measures and Patient Education

Both specialties endorsed smoking cessation programs, a key intervention for respiratory health. However, occupational physicians recommended environmental interventions at a much higher rate than pulmonologists. This aligns with their core focus on primary prevention and workplace safety compliance. Furthermore, this again reflects the core message of Nicholson et al. [15], who state, “the most important action to prevent cases of occupational asthma is to reduce exposure at source”. Patient education priorities also reflected specialty mandates. Pulmonologists focused on disease mechanisms, inhaler technique, and medication adherence, consistent with optimizing clinical outcomes. Occupational physicians emphasized hazard recognition, PPE use, and early symptom reporting, aligning with exposure control and regulatory responsibilities [6].

### 4.4. Interdisciplinary Collaboration

Our findings suggest a consensus between specialties that interdisciplinary collaboration is a frequent and necessary part of ORD management, with a similar percentage of both groups endorsing this view. This shared understanding provides a strong foundation for building more integrated care models. However, it is significant that 30% of the physicians reported collaborating with other specialists only occasionally. The higher rate of “Very frequent” collaboration reported by occupational physicians (13.7%) likely reflects their role in initiating referrals and coordinating care with clinical specialists for diagnostic confirmation and medico-legal evaluations. The fact that a substantial portion of pulmonologists reported collaborating only “Occasionally” or “Rarely” suggests that formal, structured interactions may be less common than informal consultations, pointing to a key gap where formalized communication protocols are needed. This fragmented model mirrors findings from previous studies, where a lack of integration between clinical and occupational services led to delayed diagnosis, incomplete exposure mitigation, and suboptimal health outcomes [4,14,15].

### 4.5. Barriers to Optimal Management

The results concerning “Preventive interventions recommended to patients and employers”, “Patient education topics addressed during consultations”, “Tools and methods for monitoring disease progression” and “Perceived systemic and operational barriers to optimal ORD management” were not statistically significant. The specialists selected multiple, often similar options, consequently limiting the ability to identify significant differences. In particular, a critical issue found within the obstacles to optimal management is the limited access to specialized diagnostic tools. This finding may reflect broader systemic challenges within the Italian national healthcare system that can create disparities in resource availability for practitioners, hindering timely and accurate diagnosis. Furthermore, almost all physicians recognized inadequate patient adherence and lack of standardized guideline as the main obstacles.

### 4.6. Strengths and Limitations

The strengths of this study include its national scope, balanced representation of two key specialties, and alignment of the questionnaire with established guidelines and European studies, ensuring relevance and generalizability.

This study has several limitations that should be acknowledged. First, our recruitment strategy of targeting members of national professional societies may introduce a selection bias, as the practices of physicians who are not members of these organizations could differ. Second, the self-reported nature of the data carries a risk of response bias; participants may have described idealized practices aligned with guidelines rather than their actual, day-to-day behaviors, a point underscored by the absence of objective validation through chart reviews.

Another key limitation of our study is its statistical power. A post hoc power analysis indicated that a sample size of approximately 132 participants would be required to reliably detect a medium effect size with 80% power. Our final sample of 102 participants was therefore slightly underpowered, which increases the risk of a Type II error (i.e., a false negative). Consequently, non-significant findings should be interpreted with caution, and future research in this area would benefit from larger, multi-center cohorts to confirm our results.

Furthermore, the questionnaire employed the general term ‘Pulmonary Function Test’ without differentiating between simple spirometry and more comprehensive global spirometry, which includes techniques like body plethysmography or gas dilution methods. Consequently, our data reflect the overall use of PFTs and cannot be used to determine the specific utilization rates of these advanced diagnostic techniques, as they are aggregated within this broader category.

Additionally, a limitation of this study is that the psychometric properties of the survey instrument, such as internal consistency and test–retest reliability, were not formally assessed. While the questionnaire was developed based on a thorough literature review and expert consensus, future studies should utilize a formally validated instrument to corroborate these findings.

Finally, our findings are contextualized within the unique structure of the Italian healthcare system. The specific roles, training pathways, and regulatory frameworks for pulmonologists and occupational physicians in Italy mean that the disciplinary gaps and collaborative challenges identified may not be directly generalizable to other countries with different healthcare models. Future research could explore these dynamics in other national contexts.

### 4.7. Clinical and Policy Implications

These findings underscore the urgent need for integrated care pathways that combine clinical diagnosis and pharmacotherapy with workplace exposure assessment and preventive interventions. Developing shared national guidelines, endorsed jointly by pulmonology and occupational medicine societies, could harmonize practices, improve early diagnosis, and optimize patient outcomes.

Interdisciplinary training during residency programs and continuing medical education workshops may foster mutual understanding, clarify roles, and facilitate structured referral pathways. Hospitals and occupational health units should establish formalized communication protocols, including joint case discussions and shared electronic health records, to bridge current gaps [15].

While this analysis has focused on the divergences, it is crucial to recognize the substantial areas of overlap and shared principles that provide a strong foundation for integration. For instance, both specialties demonstrate a fundamental agreement on the initial steps of patient evaluation, prioritizing the patient interview and a detailed work and exposure history. This shared diagnostic starting point is a critical asset. Furthermore, there is perfect alignment on key preventive measures like recommending smoking cessation (both 44.1%) and a mutual recognition of systemic barriers, such as poor patient adherence and limited access to advanced tools. These points of convergence are not trivial; they represent a shared philosophy and a common understanding of the challenges, which are prerequisites for building the effective, integrated care pathways we recommend.

### 4.8. Research Recommendations

To address the challenges highlighted by our study, we recommend the following:

1. Development of integrated national guidelines, jointly endorsed by pulmonology and occupational medicine societies, outlining standardized diagnostic algorithms, treatment strategies, and preventive measures for ORDs;
2. Implementation of structured interdisciplinary pathways, incorporating formalized referral protocols, joint case discussions, and shared electronic health records to facilitate efficient communication and co-management;
3. Inclusion of interdisciplinary training modules within residency programs and continuing medical education to enhance mutual understanding of each specialty’s role and foster collaborative practice;
4. Strengthening occupational health surveillance systems to ensure early identification of work-related respiratory hazards and prompt intervention to prevent irreversible disease progression.

Finally, future multicenter prospective studies are needed to evaluate the impact of integrated care models on clinical outcomes, work ability preservation, and health system costs, while incorporating patient-reported experiences to inform person-centered occupational respiratory disease management.

## 5. Conclusions

As the first nationwide comparative survey of its kind in Italy, this study provides novel insights into the management of occupational respiratory diseases (ORDs), revealing that despite a shared goal, pulmonologists and occupational physicians adopt fundamentally divergent approaches. These divergences, however, build upon a shared foundation of core clinical practices, including a universal emphasis on smoking cessation and the use of spirometry for disease monitoring. Pulmonologists focus primarily on clinical diagnosis and pharmacological management, guided by international recommendations such as GINA and GOLD, to achieve symptom control, prevent disease progression, and optimize lung function. In contrast, occupational physicians prioritize workplace exposure assessment, risk reduction interventions, and preventive strategies, reflecting their public health and regulatory responsibilities.

The study highlights critical challenges, including fragmented interdisciplinary communication, limited integration of diagnostic and preventive pathways, and the absence of shared national guidelines that harmonize clinical and occupational management. These gaps may lead to delayed diagnosis, suboptimal treatment, and inadequate exposure mitigation, ultimately compromising patient outcomes and occupational health protection.

Bridging these disciplinary gaps is essential to deliver effective, holistic, and preventive care to workers affected by or at risk of occupational respiratory diseases, safeguarding both their health and their livelihoods.

## Figures and Tables

**Table 1:** Data results for categorical variables. * Statistical significance set at *p* < 0.05.

	Item	*p*-Value
1	Initial evaluation methods	0.004 *
2	Diagnostic tools	0.0003 *
3	Diagnostic criteria	0.01 *
4	First-line therapeutic strategies	0.001 *
5	Frequency and structure of follow-up visits	0.00001 *
6	Preventive interventions recommended to patients and employers	0.327
7	Frequency and modality of interdisciplinary collaboration	0.002 *
8	Patient education topics addressed during consultations	0.185
9	Tools and methods for monitoring disease progression	0.340
10	Perceived systemic and operational barriers to optimal ORD management	0.650

**Table 2:** Participant characteristics.

	Pulmonologists	Occupational Physicians
N	51	51
Mean age (years)	47.5 ± 9.2	50.1 ± 8.4
Average clinical experience (years)	18	20
Work setting	Tertiary care hospital (62%)	Territorial occupational health units (72%)
	Secondary level public hospital (28%)	Large industries (18%)
	Private practice (10%)	Private consultancy (10%)

**Table 3:** Initial evaluation methods.

	Patient Interview and Physical Examination	Review of the Patient’s Work History	Spirometry	Imaging	Other
Pulmonologists	50	1	0	0	0
Occupational physicians	39	8	2	1	1

**Table 4:** Diagnostic Tools. XR, X-Rays; CT, Computed Tomography; MRI, Magnetic Resonance Imaging.

	Pulmonary Function Test	Imaging (XR, CT, MRI)	Bronchoscopy	Allergologic Tests	Detailed Occupational and Exposure History
Pulmonologists	35	12	1	1	2
Occupational physicians	20	15	0	0	16

**Table 5:** Diagnostic criteria.

	Symptom Presentation	Work History and Risk Exposure	Pulmonary Function Test Results	Imaging Results	Biopsy Results
Pulmonologists	36	42	55	42	3
Occupational physicians	28	41	31	33	14

**Table 6:** First-line therapeutic strategies.

	Pharmacotherapy	Environmental Control and Risk Exposure	Rehabilitation Therapy	Patient Self-Education	Referring Patients to Other Specialist
Pulmonologists	28	15	3	3	2
Occupational physicians	13	16	3	3	16

**Table 7:** Frequency and structure of follow-up visits.

	Weekly	Monthly	Three-Monts	Six-Months	Yearly
Pulmonologists	1	4	9	24	3
Pulmonologists	1	4	9	24	3
Occupational physicians	0	9	9	19	14

**Table 8:** Preventive interventions recommended to patients and employers.

	Personal Protective Equipment	Environmental Interventions	Smoking Cessation	Scheduled Medical Follow-Up	B
Pulmonologists	37	25	45	33	27
Occupational physicians	45	30	45	24	41

**Table 9:** Frequency and modality of interdisciplinary collaboration.

	Very Frequently	Frequently	Occasionally	Rarely	Never
Pulmonologists	2	24	18	7	0
Pulmonologists	2	24	18	7	0
Occupational physicians	14	22	13	1	1

**Table 10:** Patient education topics addressed during consultations.

	Disease Mechanisms	Therapy Adherence Importance	Exposure Avoidance	Early Symptom Reporting	Lifestyle Changes
Pulmonologists	46	46	40	12	32
Occupational physicians	35	32	33	5	39

**Table 11:** Tools and methods for monitoring disease progression.

	Regular Follow-Up Appointments	Pulmonary Function Test	Symptom Diaries	Imaging	Blood Tests
Pulmonologists	45	46	11	32	10
Occupational physicians	31	37	18	29	12

**Table 12:** Perceived systemic and operational barriers to optimal ORD management.

	Poor Patient Adherence	Limited Access to Advanced Diagnostic Tools	Insufficient Educatonal Material	Challenges in Multidisciplinary Material	Other
Pulmonologists	52	24	9	16	0
Occupational physicians	43	28	7	12	1

## Data Availability

The original contributions presented in this study are included in the article. Further inquiries can be directed to the corresponding author.
